# Epigenome-wide association study in hepatocellular carcinoma: Identification of stochastic epigenetic mutations through an innovative statistical approach

**DOI:** 10.18632/oncotarget.17462

**Published:** 2017-04-27

**Authors:** Davide Gentilini, Stefania Scala, Germano Gaudenzi, Paolo Garagnani, Miriam Capri, Matteo Cescon, Gian Luca Grazi, Maria Giulia Bacalini, Serena Pisoni, Alessandra Dicitore, Luisa Circelli, Sara Santagata, Francesco Izzo, Anna Maria Di Blasio, Luca Persani, Claudio Franceschi, Giovanni Vitale

**Affiliations:** ^1^ Istituto Auxologico Italiano IRCCS, Cusano Milanino, Milan, Italy; ^2^ Functional Genomics, Istituto Nazionale per lo Studio e la Cura dei Tumori, IRCCS Fondazione “G. Pascale”, Napoli, Italy; ^3^ Department of Clinical Sciences and Community Health (DISCCO), University of Milan, Milan, Italy; ^4^ Department of Experimental, Diagnostic and Specialty Medicine, Alma Mater Studiorum, University of Bologna, Bologna, Italy; ^5^ Interdepartmental Center “L. Galvani”, University of Bologna, Bologna, Italy; ^6^ DIMEC-Department of General Surgery and Medicine Sciences, S. Orsola-Malpighi Hospital, Bologna, Italy; ^7^ Regina Elena National Cancer Institute Via Elio Chianesi 53, Rome, Italy; ^8^ IRCCS Institute of Neurological Sciences, Bologna, Italy; ^9^ Department of Experimental Oncology, Laboratory of Molecular Biology and Viral Oncology, Istituto Nazionale per lo Studio e la Cura dei Tumori, Fondazione “G. Pascale”, Napoli, Italy; ^10^ Department of Surgical Oncology, Abdominal and Hepatobiliary Unit, Istituto Nazionale per lo Studio e la Cura dei Tumori, IRCCS Fondazione “ G. Pascale”, Napoli, Italy

**Keywords:** hepatocellular carcinoma, stochastic epigenetic mutation, epigenetics, DNA methylation, genome-wide

## Abstract

Hepatocellular carcinoma (HCC) results from accumulation of both genetic and epigenetic alterations. We investigated the genome-wide DNA methylation profile in 69 pairs of HCC and adjacent non-cancerous liver tissues using the Infinium HumanMethylation 450K BeadChip array. An innovative analytical approach has been adopted to identify Stochastic Epigenetic Mutations (SEMs) in HCC.

HCC and peritumoral tissues showed a different epigenetic profile, mainly characterized by loss of DNA methylation in HCC. Total number of SEMs was significantly higher in HCC tumor (median: 77,370) than in peritumoral (median: 5,656) tissues and correlated with tumor grade. A significant positive association emerged between SEMs measured in peritumoral tissue and hepatitis B and/or C virus infection status. A restricted number of SEMs resulted to be shared by more than 90% of HCC tumor samples and never present in peritumoral tissue. This analysis allowed the identification of four epigenetically regulated candidate genes (AJAP1, ADARB2, PTPRN2, SDK1), potentially involved in the pathogenesis of HCC.

In conclusion, HCC showed a methylation profile completely deregulated and very far from adjacent non-cancerous liver tissues. The SEM analysis provided valuable clues for further investigations in understanding the process of tumorigenesis in HCC.

## INTRODUCTION

Hepatocellular carcinoma (HCC) is a common cancers and the second leading cause of cancer-related death among males in the world [1]. Indeed, advanced HCC not eligible for local therapies has limited chances of cure [2], notwithstanding a massive effort in identifying novel therapeutic targets and predictive markers [3–5]. The pathogenesis of HCC is multifactorial and includes several extrinsic and intrinsic factors. Chronic liver damage secondary to viral hepatitis, alcohol abuse or non-alcoholic steatohepatitis, represents the most critical determinant in the development of this tumor. Several recent studies have provided relevant insights in this field through the characterization of HCC by using whole genome/exome sequencing, genome-wide methylation arrays and RNA sequencing [6–8]. Although it is known that HCC results from accumulation of both genetic and epigenetic alterations, the molecular pathogenesis of this tumor is still not completely understood. Indeed, HCC is a complex and heterogeneous disease [9].

DNA methylation is one of the most widely characterized epigenetic modifications implicated in cancer development. Several studies evaluated the DNA methylation profile in HCC through genome-wide arrays [10–14]. Most of these reports compared the mean methylation level calculated for each CpG site in human HCC with that in adjacent non-cancerous liver tissue, and correlated the tumor methylation profile with clinical and pathological parameters in order to obtain a deeper understanding of the pathogenesis of this tumor. While this approach allows to identify epigenetic alterations shared by a group of subjects and potentially associated with their phenotype, rare or stochastic epigenetic alterations, that are not shared among subjects and minimally affect the mean methylation level of the group, remain unexplored, although they may play a role in hepatocarcinogenesis. In addition, an analytical strategy based on comparisons of mean methylation values does not allow to process data obtained from a single subject [15].

Epigenetic mutations, or the more common term “epimutations” are heritable changes in gene activity not associated with a DNA mutation but rather with gain or loss of DNA methylation or other inheritable modifications of chromatin [16]. We have recently developed an analytical approach able to identify stochastic epigenetic mutations (SEMs) not shared among subjects. We applied this peculiar analytical strategy to investigate the relations between stochastic epigenetic alterations and aging [15].

In the present study, we investigated the genome-wide DNA methylation profile in HCC, adjacent non-cancerous liver tissue and normal liver tissue. A new analytical approach has been adopted to identify epimutations potentially involved in the development of HCC.

## RESULTS

### Clinical and pathological characteristics of HCC patients

Clinical and pathological characteristics of enrolled HCC patients are described in Table 1. The mean age at HCC diagnosis was 65.9 ± 8.8 years. Most patients were male (76.8%) and positive for HCV (59.5%). Among the 69 HCC patients, 87% have pathologically defined cirrhosis and 85.6% have tumors grade II.

**Table 1:** Clinical and pathological characteristics of 69 HCC patients

Variables	Number of cases (%)
**Age**	
< 60 years	17 (24.6)
> 60 years	52 (75.4)
**Gender**	
Male	53 (76.8)
Female	16 (23.2)
**Viral infection**	
No Viral Infection	15 (21.8)
HBV	9 (13)
HCV	41 (59.5)
HBV and HCV	4 (5.7)
**Alcoholic liver disease**	
Yes	3 (4.4)
No	66 (95.6)
**Non-alcoholic steatohepatitis**	
Yes	8 (11.5)
No	61 (88.5)
**Cirrhosis**	
Yes	60 (87)
No	9 (13)
**Child-Pugh class**	
A	57 (82.6)
B	12 (17.4)
**Tumor Grade**	
I	3 (4.2)
II	59 (85.6)
III	7 (10.2)
**Tumor Number**	
1	49 (71)
2	11 (15.9)
3	4 (5.7)
4	5 (7.4)

The complexity of variability of all the phenotypic traits has been reduced using Multiple Factor Analysis (Supplementary Figure 1). The first and second dimensions explained 18.29% and 16.63%, respectively, of the total variability of phenotypic traits.

### Genome-wide methylation profiles in HCC tumor and adjacent non-cancerous tissues

**Figure 1:**
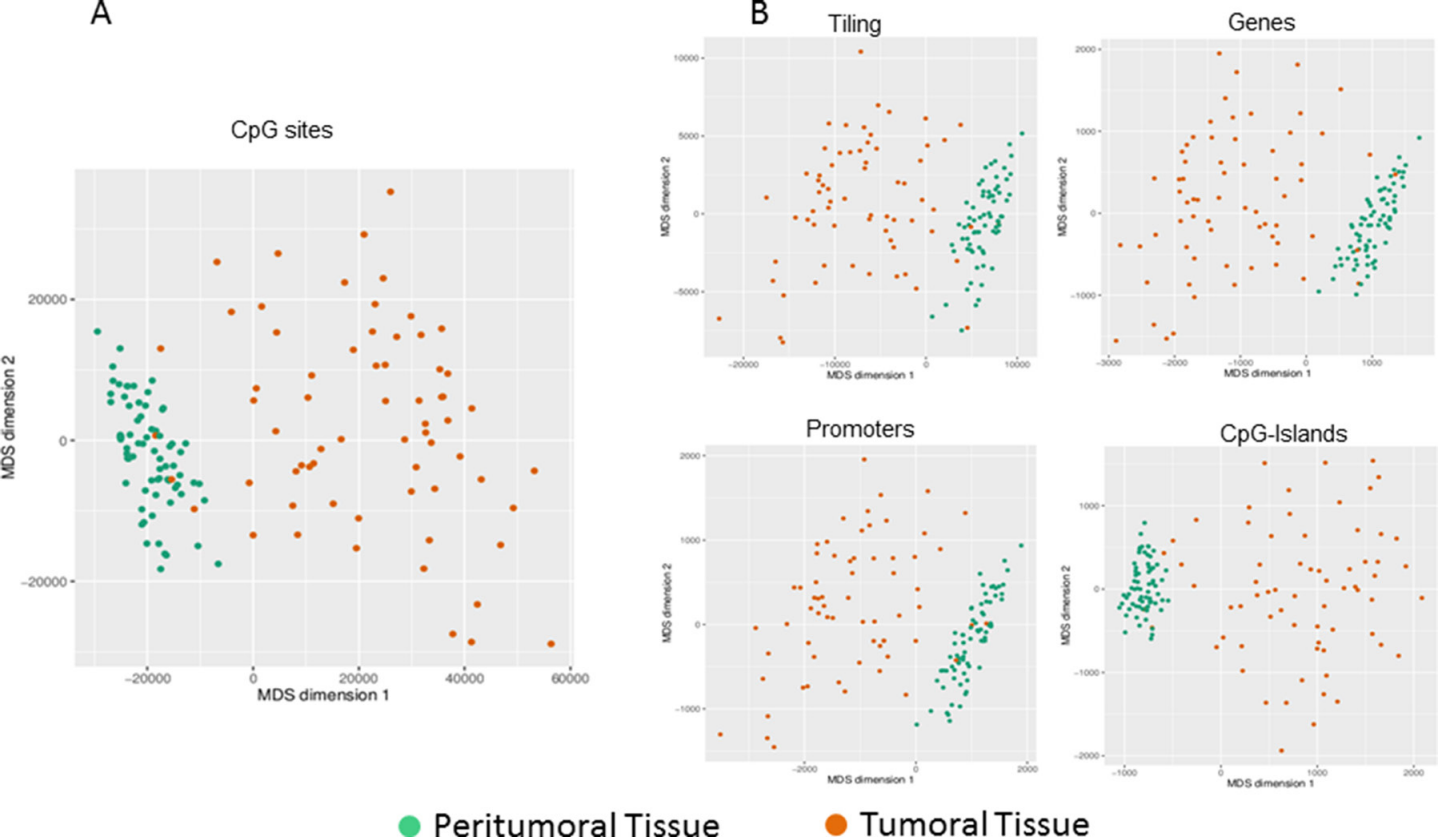
Exploratory analysis of methylation data Dimension reduction obtained by Multi Dimensional Scaling (MDS) is used to visually resume the complexity of methylation profile in HCC and peritumoral tissues at site level (**A**) and at regional level (**B**). Plots have been obtained using the first two dimensions.

**Figure 2:**
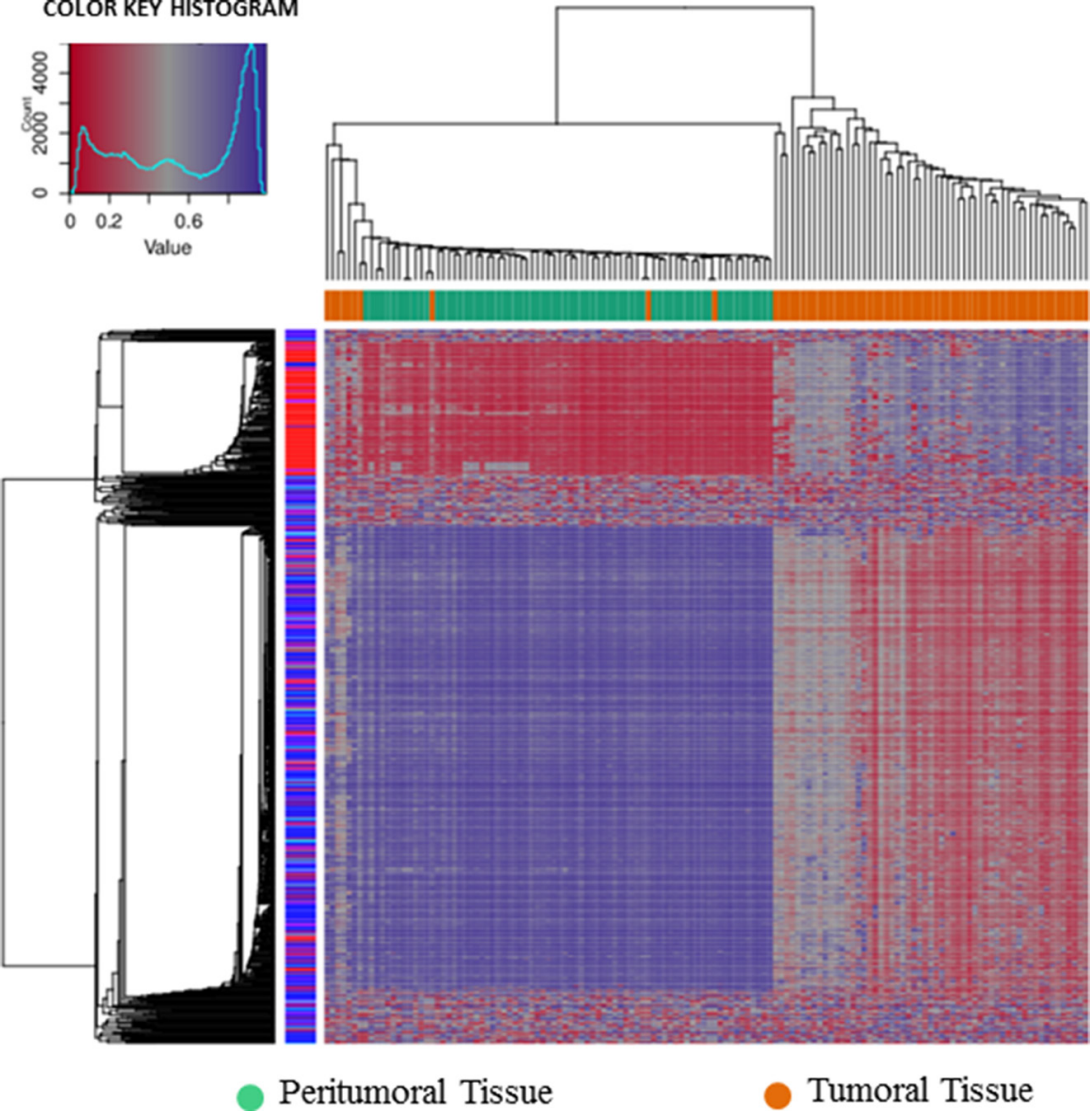
Hierarchical cluster analysis Hierarchical cluster analysis of the top 1000 significantly differentially methylated cpG sites between 69 pairs of HCC and adjacent non-cancerous liver tissues.

A dimension reduction approach was used to visually inspect the dataset for signals in the methylation values. The Multi Dimensional Scaling Analysis was performed considering methylation signals from all CpG sites (Figure 1A) and in several genomic regions (tiling, genes, promoters and CpG Islands) (Figure 1B). This analysis showed a dramatic difference in methylation levels between HCC and peritumoral tissues. The Figure 2 shows hierarchical clustering of samples based on the top 1,000 CpG loci differentially methylated between HCC tumor and adjacent tissues. Excellent separation of tumor and peritumoral non-cancerous tissues was observed with only 3 misclassifications.

**Figure 3:**
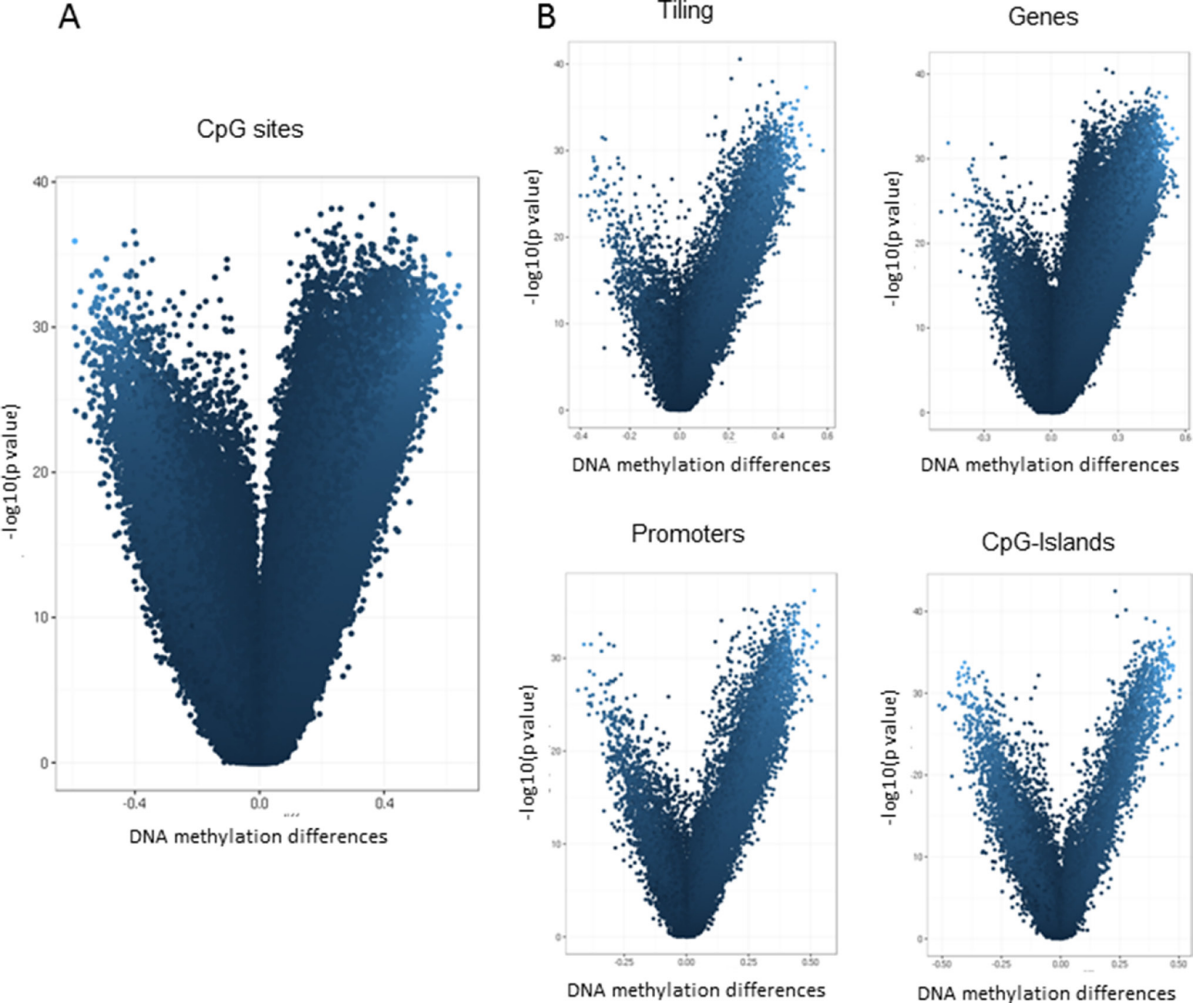
Differential methylation analysis of Illumina 450K data Volcano plot showing differential methylation analysis of 69 paired HCC tumor and adjacent non-cancerous liver tissues at site level (**A**) and at regional level (**B**). The x-axis represents the mean DNA methylation difference (adjacent non-cancerous liver tissue – HCC), while the y-axis shows the – log10 of the *p value* for each CpG site (A) or genomic region (B).

Paired differential methylation analysis between HCC *vs* peritumoral tissues was computed across the entire epigenome and selecting specific regions. The overall analysis identified 302,133 CpG sites out of 473,929 across the entire epigenome that significantly differed in methylation levels. Among all significant CpG sites, 36,615 probes resulted to have a delta greater than |0.30|. Differential methylation at the region level was computed based on a variety of metrix and combining methylation values inside tiling, genes, promoters and CpG Islands. Considering all the subgroups, the number of regions that resulted significantly different between the two sets and the number of the regions included in the array were: 105,827 and 131,743 for tiling, respectively; 25,572 and 29,592 for genes, respectively; 22,827 and 29,740 for promoters, respectively; 17,534 and 25,837 for CpG islands, respectively. Mean DNA methylation differences are represented as a volcano plot in Figure 3. Scatterplots for differential methylation at site and regional levels are shown in Supplementary Figure 2.

These data indicate that aberrant DNA methylation in HCC is a very common event, covering the entire epigenome and mainly characterized by a loss of methylation in the tumor tissue. Considering that methylation differences between the two groups resulted extremely strong and involved the whole epigenome, a prioritization of significant results has been performed. The top 10 hypomethylated or hypermethylated genes extracted from this analysis and ranked by statistical significance are reported in Table 2.

**Table 2:** Top 10 ranked hypomethylated or hypermethylated genes in HCC tumor tissue obtained after prioritization of all differentially methylated probes

Hypomethylated genes									
						Mean Methylation values			
Gene Symbol	Ensemble_ID	Chromosome	Start	End	Number of CpG sites	Peritumoral Tissue	Tumor Tissue	Delta	Adjusted *P* Value
IGF1R	ENSG00000140443	chr15	99192200	99507759	129	0.698	0.449	0.24	1.39E-22
HGF	ENSG00000019991	chr7	81328322	81399754	9	0.433	0.237	0.19	1.66815E-17
HLA-DQA1	ENSG00000196735	chr6	32595956	32614839	8	0.583	0.341	0.24	1.49596E-19
PIK3CG	ENSG00000105851	chr7	106505723	106547590	12	0.736	0.473	0.26	2.92839E-19
MAPK10	ENSG00000109339	chr4	86936276	87515284	40	0.638	0.449	0.18	1.14679E-19
KDR	ENSG00000128052	chr4	55944644	55991756	9	0.392	0.270	0.12	5.39177E-13
FGFR1	ENSG00000077782	chr8	38268656	38326352	29	0.407	0.365	0.04	2.63192E-12
GNGT2	ENSG00000167083	chr17	47280153	47287936	11	0.587	0.388	0.19	3.49546E-17
PLCB4	ENSG00000101333	chr20	9049410	9461889	8	0.618	0.355	0.26	6.88013E-21
ADCY2	ENSG00000078295	chr5	7396321	7830194	29	0.667	0.363	0.30	9.6768E-27

Hypermethylated genes									
						Mean Methylation values			
Gene Symbol	Ensemble_ID	Chromosome	Start	End	Number of CpG sites	Peritumoral Tissue	Tumor Tissue	Delta	Adjusted *P* Value
APC	ENSG00000134982	chr5	112043195	112181936	25	0.307	0.596	−0.28	1.76369E-20
HIST1H4F	ENSG00000198327	chr6	26240561	26240976	4	0.140	0.482	−0.34	1.50784E-24
FGF19	ENSG00000162344	chr11	69513000	69519410	19	0.194	0.434	−0.23	5.56857E-18
FZD1	ENSG00000157240	chr7	90893783	90898123	9	0.321	0.557	−0.23	7.55218E-26
LPAR2	ENSG00000064547	chr19	19734477	19739739	16	0.375	0.570	−0.19	3.69839E-17
HIST1H3F	ENSG00000256316	chr6	26250370	26250835	4	0.101	0.442	−0.34	3.81087E-26
HIST1H3G	ENSG00000256018	chr6	26271146	26271612	5	0.324	0.582	−0.25	9.95446E-23
HIST1H3J	ENSG00000197153	chr6	27858093	27860884	7	0.263	0.534	−0.27	1.27291E-18
PMAIP1	ENSG00000141682	chr18	57567180	57571538	2	0.059	0.27	−0.22	3.79856E-16
RASGRF2	ENSG00000113319	chr5	80256491	80525975	30	0.696	0.712	−0.02	9.64807E-08

### Stochastic epigenetic mutation analysis

**Figure 4:**
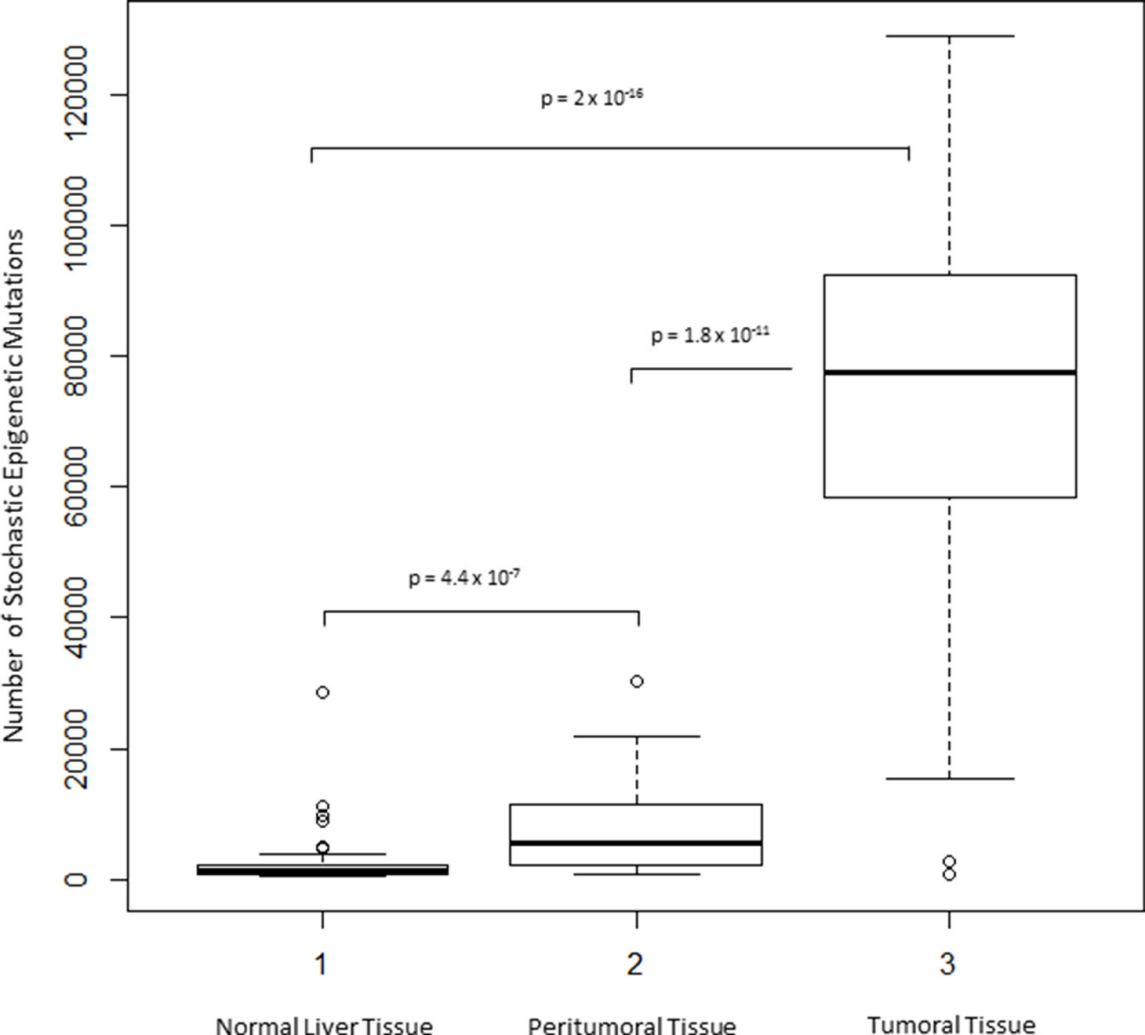
Exploration of SEMs in HCC Box Plots show distribution of SEMs number in normal, peritumoral and HCC tumor tissues.

For each sample, as described in the Material and Method section, the total number of SEMs was calculated using tissue from normal liver as reference. The number of SEMs resulted to be statistically significant higher in HCC (median: 77,370, IQR: 58,260–92,220) compared to adjacent non-cancerous tissue (median: 5,656, IQR: 2,068–7,431, *p* = 1.8 × 10^−11^) and normal liver tissue extracted from healthy subjects (median: 1,308, IQR: 854-2,148, *p* = 2 × 10^−16^) (Figure 4). Chromosomal distribution of SEMs in HCC samples and adjacent non-cancerous tissue has been reported in Supplementary Figure 3.

### Associations between SEMs and clinical and pathological characteristics

A multivariate regression analysis was performed in order to identify clinical and pathological traits potentially associated with SEMs. The number of SEMs identified in HCC and adjacent non-tumor tissue has been used as dependent variable, while specific phenotypic traits (viral infections, alcoholic liver disease, non-alcoholic steatohepatitis, cirrhosis, Child-Pugh class, tumor grade, tumor number and Multiple Factor Analysis dimensions) have been adopted as independent variables. All the multiple regression models have been adjusted for age.

**Figure 5:**
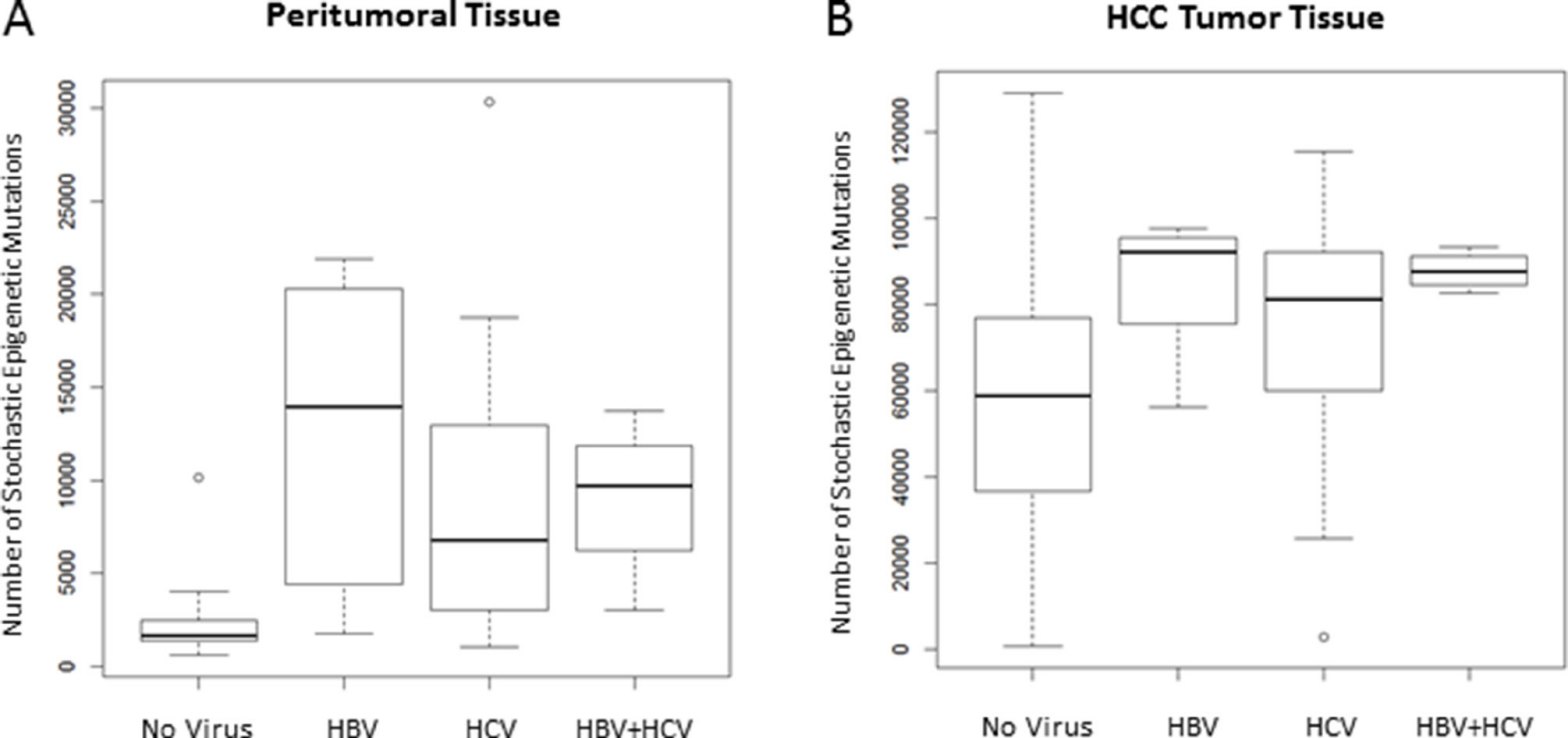
Number of SEMs is associated to viral infection Box Plots show distribution of SEMs in peritumoral tissue (**A**) and HCC tumor tissue (**B**) in relation to the HBV/HCV infection status.

Viral infection resulted significantly associated to the number of SEMs observed in peritumoral tissue. In the multivariate regression hepatitis B virus (HBV), hepatitis C virus (HCV) and HBV+HCV infection status resulted independently associated to the number of SEMs observed in peritumoral tissue (*p* = 8 × 10^−5^, *p* = 0.002 and *p* = 0.027, respectively) and, less pronounced, in tumor (*p* = 0.032, *p* = 0.034 and *p* = 0.075, respectively). Figure 5 shows the number of SEMs observed in peritumoral tissue (Figure 5A) and in HCC tumor tissue (Figure 5B) according to the viral infection status.

**Figure 6:**
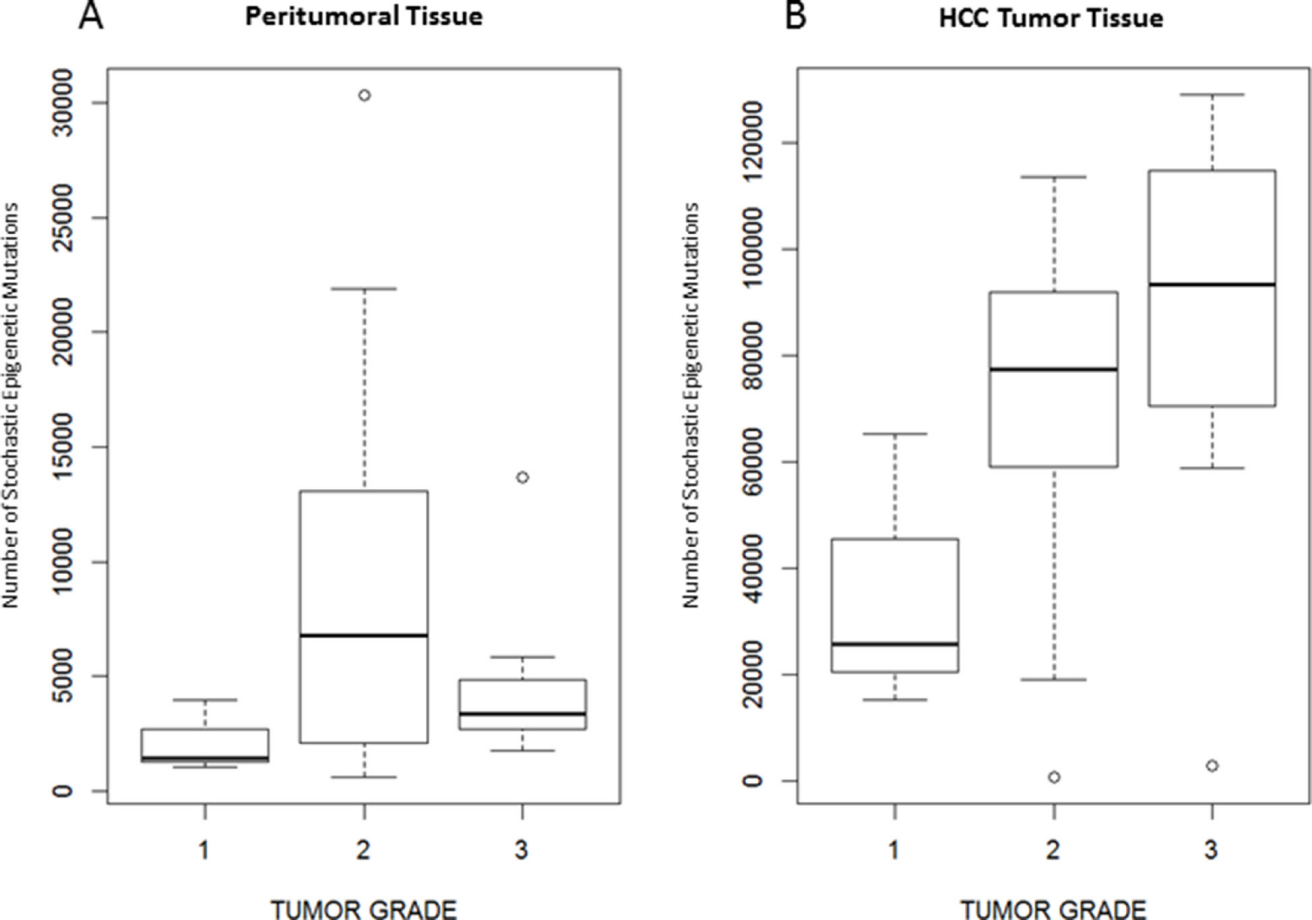
Number of SEMs is associated to tumor grade Box Plots showing distribution of SEMs in peritumoral tissue (**A**) and tumoral tissue (**B**) in relation to the tumor grade.

Tumor grade resulted significantly associated to the number of SEMs observed in HCC tumor tissue (p = 0.02), but not in peritumoral tissue (p = 0.40). Figure 6 shows the number of SEMs observed in peritumoral tissue (Figure 6A) and in HCC tumor tissue (Figure 6B) according to the tumor grade.

Complexity of clinical traits has been initially reduced using the Multiple Factor Analysis. The correlogram in Supplementary Figure 1 shows clinical traits associated to each dimension. The multiple regression analysis indicated that dimensions 1 (*p* = 2 × 10^−7^) and 5 (*p* = 1.6 × 10^−5^) resulted associated to SEMs observed in HCC tumor, while only dimension 3 (mainly collecting variability of age, HBV, HBV+HCV and non-alcoholic steatohepatitis) resulted significantly associated to the number of SEMs observed in HCC tumor and peritumoral tissues (*p* = 0.0003 and *p* = 0.015, respectively).

For other phenotypic traits the associations with the number of SEMs were not significant.

### SEMs annotation analysis and candidate markers

Number of SEMs has been calculated for all samples. We identified 109,174 and 14,315 different SEMs in HCC tumor and adjacent non-tumor tissues, respectively. Among them 101,921 SEMs resulted present only in HCC and absent in adjacent non-cancerous tissues. We annotated genomic position of SEMs discovered in each sample and selected SEMs that were present in more than 90% of HCC samples and never present in adjacent non-cancerous tissues. Through this procedure a list of 4 genes (Table 3) was identified: Adherens Junctions Associated Protein 1 (AJAP1), Adenosine Deaminase RNA Specific B2 (ADARB2), Protein Tyrosine Phosphatase Receptor Type N2 (PTPRN2) and Sidekick Cell Adhesion Molecule 1 (SDK1).

**Table 3:** List of genes epimutated in more than 90% of HCC samples and never found in peritumoral tissues

Gene Symbol	Ensemble_ID	Chr	Start	End	Promoter Mean Methylation Level Normal Liver Tissue	Promoter Mean Methylation Level Peritumoral Tissue	Promoter Mean Methylation Level Tumoral Tissue	Gene Body Mean Methylation Level Normal Liver Tissue	Gene Body Mean Methylation Level Peritumoral Tissue	Gene Body Mean Methylation Level Tumoral Tissue
AJAP1	ENSG00000196581	chr1	4714792	4852594	0.12	0.11	0.28	0.6	0.65	0.37
ADARB2	ENSG000000185736	chr10	1228073	1779670	0.08	0.08	0.12	0.72	0.75	0.41
PTPRN2	ENSG00000155093	chr7	157331750	158380480	0.25	0.28	0.45	0.8	0.77	0.43
SDK1	ENSG00000146555	chr7	3341080	4308632	0.12	0.1	0.36	0.85	0.81	0.49

## DISCUSSION

DNA methylation is the main epigenetic mechanism adopted by cells to regulate gene expression, and alterations of this mechanism are involved in the pathogenesis of cancer. In this paper, we studied DNA methylation aberrations in HCC through the Illumina 450K DNA methylation array and adopting an innovative analysis devoted to the identification of epimutations.

We characterized DNA methylation profiles in paired HCC tumor and adjacent non-cancerous liver tissue samples from 69 patients. Visual inspection of DNA methylation levels, obtained through multi-dimensional scaling, revealed a marked difference between HCC tumor and peritumoral tissues. Also the differential methylation analysis confirmed substantial changes of DNA methylation mainly due to loss of methylation in HCC.

Nishida et al. [17] compared DNA-methylation profile extracted from 59 HCC with that from 58 adjacent non-cancerous liver tissues, using HumanMethylation450 BeadChip array. They identified 38,330 CpG sites differentially methylated with an effect greater than 15% between both groups of samples, of which 92% and 8% were respectively hypomethylated (mainly located within intergenic regions) and hypermethylated (predominantly observed within promoter regions and CpG islands) in HCC. A significant association between methylation profile and tumor size has been detected, particularly in HCV-positive patients. A predominant DNA hypomethylation in HCC has been also reported by several other studies and a specific subset of CpG sites correlated with HCV infection and liver cirrhosis [10–13].

In the present study we confirmed that HCC and adjacent non-cancerous tissues have a different epigenetic profile, indeed the number of differentially methylated probes resulted extremely high (n=302,133). In this condition it is difficult to identify candidate epigenetic signatures and to highlight specific biological pathways and functions, considering that epigenetic deregulation is widespread throughout the genome. Several studies selected the top hypermethylated or hypomethylated genes on the basis of *p value* or effect size [10–13]. However it has to be considered that the identification of best associations, based only on deserving of statistical thresholds, may lead to inconclusive or biased results.

On reviewing the literature, we identified four studies [10–13] that explored genome-wide DNA methylation profiles in HCC tumor and adjacent non-tumor tissues through Illumina's 450K or 27K arrays and published a list of the top differentially methylated CpG sites. A total number of 161 probes resulted combining the lists from these four studies (Supplementary Table 1). Although, all these probes have been confirmed in the present study with a similar trend, only 8 probes have been reported by at least 2 out of 4 selected studies. This poor overlap suggests that the selection of candidate markers obtained by fixing arbitrary statistical thresholds might produce biased results.

In our study, we used an alternative approach to identify epigenetic markers, based on epigenetic mutations analysis, as previously applied in the field of aging [15]. In each sample, SEMs have been identified using normal liver tissue methylation data as reference population. We have previously reported that the number of SEMs increases exponentially with age, and for this reason all analyses have been adjusted, considering age as covariate. Interestingly, we observed a progressive increase in the number of SEMs from normal liver, peritumoral tissue (predominantly with chronic hepatitis/cirrhosis) to HCC tissue. In HCC the median number of SEMs resulted 13 times higher than in adjacent non-cancerous liver tissue. Moreover the number of SEMs observed in peritumoral tissues was 4-fold higher than that identified in normal liver tissues obtained from healthy subjects. The number of SEMs observed in peritumoral and tumoral tissues resulted also significantly higher in samples with HBV and HCV viral infection. Both HBV and HCV infections have a main role in the development of HCC through genetic and epigenetic alterations. It has been reported that aberrant DNA methylation may be induced by direct viral activity or indirectly through related chronic liver inflammation [18]. In addition, it is well known that viral infection is able to affect DNA methyl transferase expression in HCC [19].

We also focused the attention on other clinical-phenotypic traits and we observed that the number of SEMs detected in HCC tissue increased progressively with histopathological tumor grade, suggesting that tumor progression and aggressiveness correlate with the accumulation of epigenetic alterations. This represents an interesting finding, supporting the potential use of SEMs number as a prognostic marker in HCC.

Therefore, the extreme heterogeneity and complexity among HCC epigenomes makes difficult to identify specific epigenetic profiles associated to phenotypic traits. While, the number of SEMs emerged as a new variable with high sensitivity and potentially able to summarize the degree of epigenetic alterations with a simple score.

In order to identify novel candidate potential epidrivers, after the calculation of SEMs, we analysed genomic position of SEMs discovered in each sample. We performed this analysis both for HCC and peritumoral tissues. We identified a list of SEMs that were present in more than 90% of HCC samples and never present in peritumoral tissues. Four genes emerged from this analysis: AJAP1, ADARB2, PTPRN2, SDK1. These epimutations were characterized by hypermethylation at promoters level and concomitant hypomethylation at gene body level in HCC tumor tissues (Table 3). Interestingly, a similar epigenetic signature for AJAP1, ADARB2 and PTPRN2 has been previously reported in glioblastoma [20–21]. Unfortunately, it was not possible using microarray-based method to understand if these epigenetic alterations were allele-specific.

AJAP1 is a component of adherent junctions. It interacts with E-cadherin–β-catenin complexes in polarized epithelial cells. The methylation of AJAP1 may lead to the release of β-catenin and the activation of Wnt signaling [22]. AJAP1 expression has been recently reported to be reduced in HCC compared with non-cancerous liver tissues and to be associated with hypermethylation of the AJAP1 promoter. Indeed, suppression of AJAP1 expression may represent a specific event that occurs in the final stage of the initiation of HCC [23]. Several evidences concerning the role of AJAP1 as a putative tumor suppressor genes regulated by promoter hypermethylation have been reported in cervical cancer, esophageal squamous cell carcinoma and gastric cancer [22, 24, 25]. In addition, it has been shown that AJAP1 expression was drastically reduced in glioblastoma tumors, and the loss of expression correlated with AJAP1 promoter hypermethylation and poorer survival. Interestingly, restoration of AJAP1 gene expression by transfection or demethylating agents decreased tumor cell proliferation and migration in glioblastoma cell lines [26].

ADARB2 encodes a member of the double-stranded RNA adenosine deaminase family of RNA-editing enzymes and plays a regulatory role in RNA editing. This may affect gene expression and function through the modulation of: mRNA translation, by changing codons and hence the amino acid sequence of proteins; pre-mRNA splicing, by altering splice site recognition sequences; RNA stability, by changing sequences involved in nuclease recognition; genetic stability in the case of RNA virus genomes, by changing sequences during viral RNA replication; and RNA structure-dependent activities, such as microRNA production or targeting or protein-RNA interactions [21]. The gene body region of ADARB2 resulted extremely hypomethylated in HCC tumor compared to adjacent tissues. Currently there are no data available concerning the potential role of ADARB2 in the development of HCC. However, a growing body of evidence indicates an important role of adenosine to inosine RNA editing, modulated through epigenetic mechanisms, in the pathogenesis and progression of cancer [21, 27]: 1) hypomethylation of ADARB2 has been reported as a marker of relapse in pediatric acute lymphoblastic leukemia; 2) RNA levels of the three ADAR family members (ADAR, ADARB1, and ADARB2) were significantly reduced in brain tumors, the reduction of ADARB2 correlated with the grade of malignancy of glioblastoma multiforme, the most aggressive of brain tumors, displaying a 99% decrease in ADARB2 RNA levels; 3) overexpression of ADAR and ADARB1 (an important paralog of ADARB2) in the U87 glioblastoma multiforme cell line significantly decreased proliferation rate.

PTPRN2 belongs to the protein tyrosine phosphatase family, it has an important role in vesicle-mediated secretory processes and exhibits a phosphatidylinositol phosphatase against phosphatidylinositol 4,5-diphosphate and phosphatidylinositol 3-phosphate [28]. PTPRN2 has been reported to be significant hypermethylated in squamous cell lung cancer and glioblastoma [20, 29]. PTPRN2 resulted in the top list of marker down methylated in HCC, as reported by Shen et al [10], and similarly to our results the hypomethylation of this gene was restricted to the gene body region.

SDK1 gene codes for a cell adhesion molecule with a potential role in cancer progression. This gene is responsive to androgens, resulted overexpressed and able to regulate cellular migration in prostate cancer [30]. Moreover, in whole-exome sequencing studies performed on adrenocortical carcinoma, this gene was affected by somatic mutations [31]. However, to the best of our knowledge there are not data regarding the role of this gene in the pathogenesis of HCC.

In summary, we confirmed that HCC showed a methylation profile completely deregulated and very far from adjacent non-cancerous liver tissues. An alternative genome-wide DNA methylation analytical approach, based on the identification of epimutations, represents the real novelty of this study. This statistical approach resulted a robust method to appreciate epigenetic variability among samples. Indeed, the number of SEMs observed in HCC tissue was strongly associated to several important phenotypic traits. In addition, this analysis allowed the identification of four epigenetically regulated candidate genes (AJAP1, ADARB2, PTPRN2, SDK1), potentially involved in the hepatocarcinogenesis. Future studies are required to further validate the differential expression of these genes at the RNA or protein level in HCC.

## MATERIALS AND METHODS

### Samples, patients and phenotypes

Paired HCC tumor and adjacent non-cancerous liver tissue samples were obtained from 69 patients (16 females, 53 males) who underwent surgical resection from 2008 to 2015 at the National Cancer Institute of Naples. Samples were frozen immediately after surgical removal and stored at −80°C until DNA extraction. Demographic data (age and gender), information on viral infection (HBV and HCV), clinical and pathological characteristics (alcoholic liver disease, non-alcoholic steatohepatitis, cirrhosis, Child-Pugh class, tumor grade and tumor number) were obtained from the medical records. Written informed consent was obtained from all patients. This study was approved by the Ethics Committee of the Istituto Auxologico Italiano.

### DNA preparation and infinium methylation 450K assay

DNA was extracted using the QIAamp DNA Mini kit (Qiagen GmbH,D-40724 Hilden, Germany), according to the manufacturer's instructions. Bisulfite modification of 1 μg DNA was conducted using an EZ DNA Methylation Kit (Zymo Research) according to the manufacturer's procedure. The Infinium Methylation 450K assay was performed according to Illumina's standard protocol. Six HCC/adjacent non-tumor tissue pairs were processed on the same chip to avoid chip-to-chip variation. Quality control and quantification of DNA were performed before and after bisulfite conversion.

### 450k methylation data and populations description

Methylation data analysed in the study were obtained from different datasets: 1. The Case Population: 69 HCC tumor tissues. 2. The Control Population: 69 paired adjacent non-cancerous liver tissue samples. 3. The Reference Population: Methylation raw data from 47 liver biopsies from healthy heart-beating and brain death donors with an age spanning from 13 to 90 years [32] were obtained from the Istituto Auxologico Italiano Epigenetic Database. Donors were free from known viral hepatitis and liver cirrhosis. Methylation row data from 67 normal liver tissues were obtained from the public functional genomics data repository GEO. We selected one dataset containing methylation idat files classified as normal liver tissue (GSE61446). Using these two datasets we obtained a unique reference containing 114 subjects. This population represented the reference population used for the estimation of epigenetic mutations.

### Data management, pre-processing, normalisation and quality control

Illumina Methylation 450K raw data were analysed using the RnBeads analysis software package [29]. Sites overlapping SNPs were firstly removed from the analysis (*n* = 4713) as well as probes on sex chromosomes (*n* = 11119). Probes and samples of highest impurity were removed from the dataset using the Greedycut algorithm provided in the RnBeads package. We have considered every β value to be unreliable when its corresponding detection *p-value* was not below the threshold (T = 0.05). The background was subtracted using the methylumi package (method “noob”) [33]. The signal intensity values were normalized using the SWAN normalization method, as implemented in the minfi package. In addition to CpG sites, 4 sets of genomic regions were covered in the analysis (tailing, genes, promoters and CpG Islands).

### Differential methylation analysis and prioritization analysis

Paired Differential methylation analysis was conducted both at site and region level according to the sample groups. *P*-values were computed using the limma method for the site level analysis, while a combined *p-value* was calculated from all site *p*-values for the region-based analysis [33]. After this analysis results have been ordered depending on their relevance on HCC field. Lists of hypermethylated and hypomethylated genes have been prioritized using the Phenolyzer on line tool (http://phenolyzer.wglab.org/) and using “HEPATOCELLULAR CARCINOMA” and “HEPATIC” as key word.

### Epimutation detection

In both HCC and adjacent liver tissue samples SEMs were identified, as previously described [15]. Briefly, after the pre-processing step, for all probes, the distribution and variability of methylation levels were studied using box and whiskers plots. Methylation levels of each sample were compared to those of normal liver tissues from a reference population. The same approach was applied also for each subject of the reference population. One by one each subject of the reference population was extracted and compared to the remaining N-1 subjects. At each locus, whenever the methylation level of one sample differed extremely from the rest of the population this outlier was considered as epimutated. Thus, for each locus, epimutated samples were identified as the extreme outliers when their methylation levels lay outside of Q1-(3 x IQR) and Q3+(3 x IQR). Finally, all epimutated loci were annotated in a new data matrix that allowed to calculate, for each sample, the total amount of epimutations and their genomic position. The box and whiskers plot analysis was conducted using boxplot function in the R *car* package and confirmed using the outlier function in the R *outliers* package. A validation of this analytical approach has been previously reported [15].

### Statistical analysis

The “Shapiro.test” function provided in the R package “stats” was applied to test normality among variables. The “Wilcox.test” function provided in the R package “class” was used to test differences between cases and controls groups for all non-parametric data. Considering the presence of categorical variables, the Multiple Factor Analysis was performed using the Multiple Factor Analysis of mixt data approach and the “FAMD” function provided in the R package “FactoMineR”. The univariate and multivariate linear regressions were conducted using the “Generalised Linear Model” function provided in the R “base” package. Bonferroni's correction was performed to correct for multiple testing.

## SUPPLEMENTARY MATERIALS FIGURES AND TABLES





## Abbreviations

HBV: hepatitis B virus
HCC: hepatocellular carcinoma
HCV: hepatitis C virus
IQR: Interquartile range
MDS: Multi Dimensional Scaling
SEMs: stochastic epigenetic mutations
