# Supplementary material for: Epigenome-wide association study in hepatocellular carcinoma: Identification of stochastic epigenetic mutations through an innovative statistical approach
Source: Oncotarget. 2017 Apr 27;8(26):41890–902. doi: 10.18632/oncotarget.17462 (PMC5522036; doi:10.18632/oncotarget.17462)
Supplement: Supplementary file 1 [file oncotarget-08-41890-s001.pdf]

Epigenome-wide association study in hepatocellular carcinoma: Identification of stochastic epigenetic mutations through an innovative statistical approach

Supplementary Materials

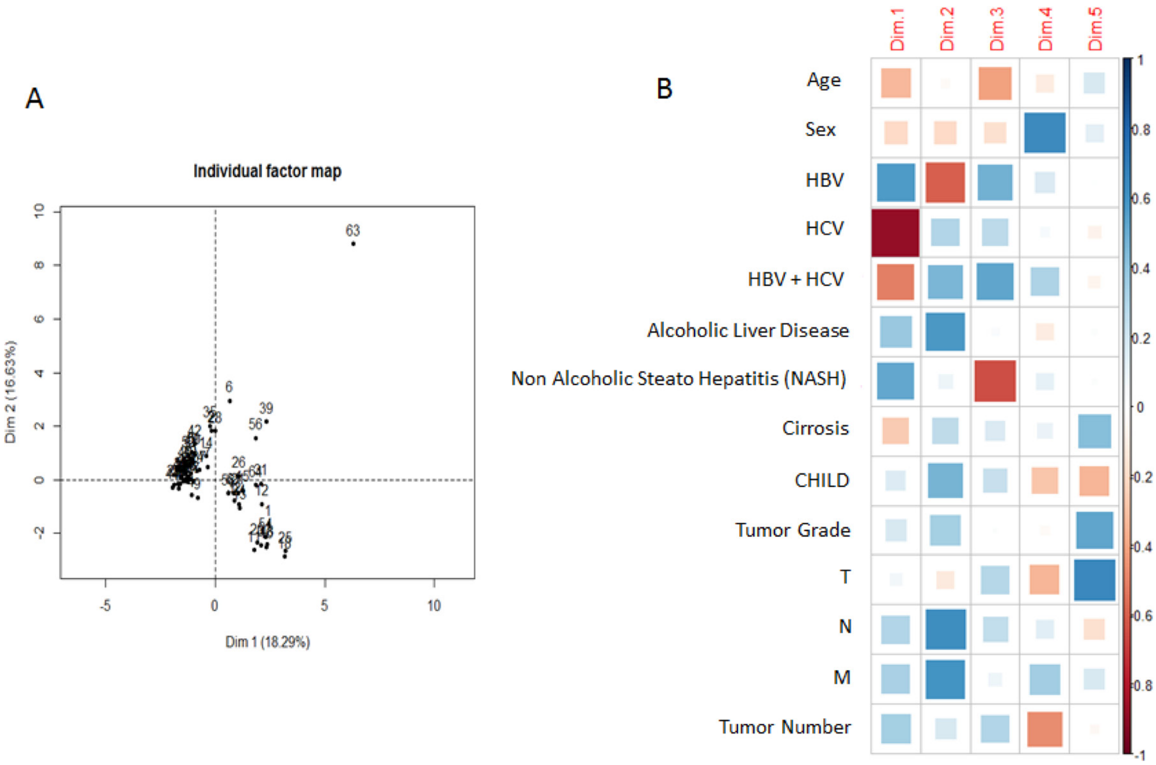

Supplementary Figure 1: Exploratory analysis of phenotypic traits. Dimension reduction obtained by Multiple Factor Analysis (MFA) is used to visually resume the complexity of phenotypic traits. The first and second dimensions explained 18.29% and 16.63% of the total variability of phenotypes, respectively. Panel (**A**) reports the distribution of subjects considering the first two dimensions. Panel (**B**) shows the correlation plot indicating phenotypic traits captured by first 5 dimensions.

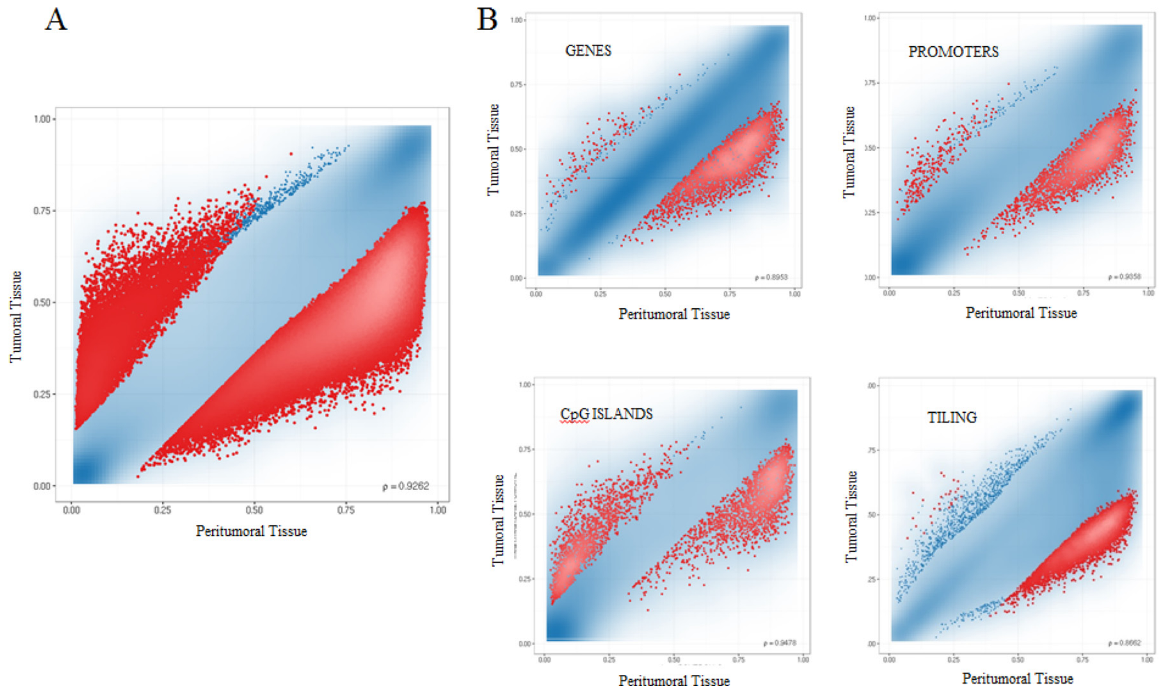

Supplementary Figure 2: Differential methylation analysis. Scatterplot for differential methylation at site Panel (**A**) and at regional levels Panel (**B**). Significantly differentially methylated probes are highlighted in red. Methylation levels of peritumoral and tumor tissues are reported on the X axis and Y axis, respectively.

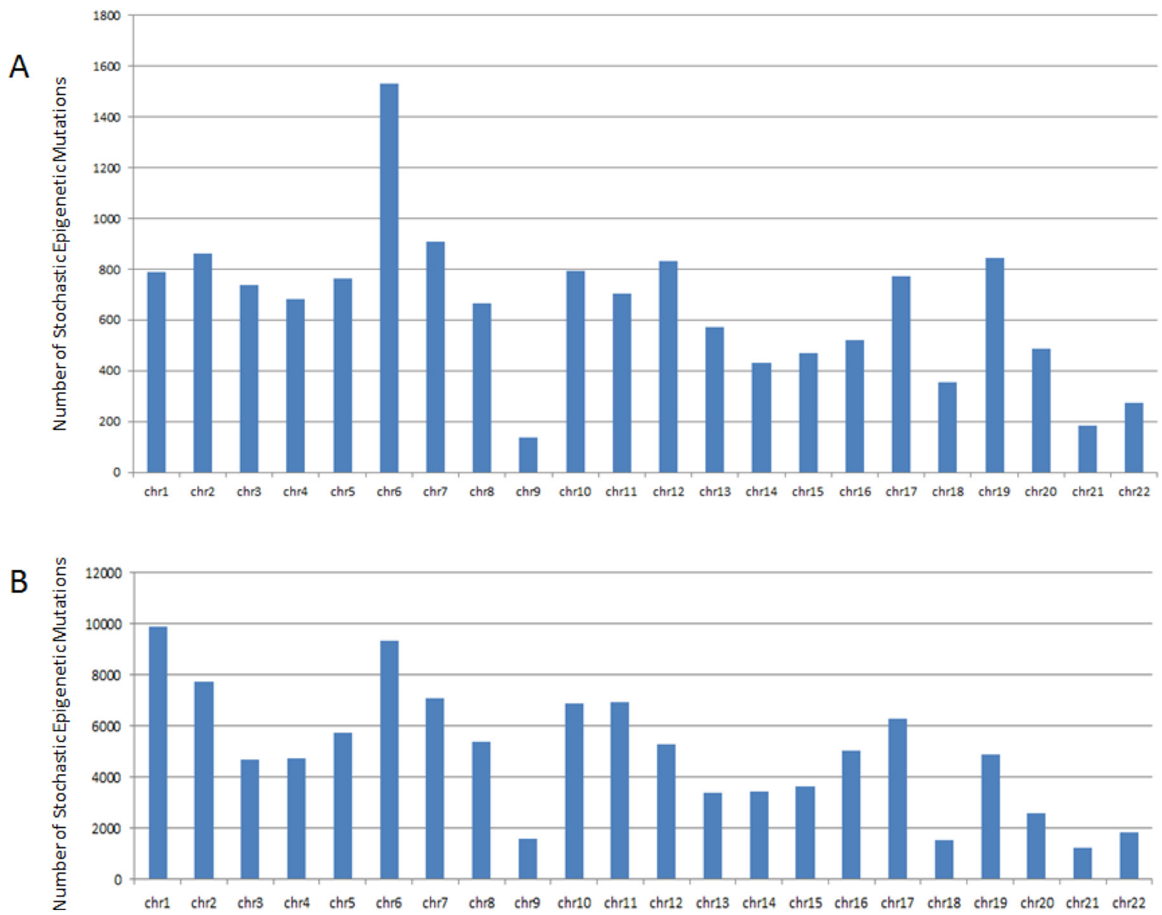

Supplementary Figure 3: Chromosomal distribution of SEMs. Chromosomal distribution of SEMs identified in Peritumoral tissues panel (**A**) and in HCC tissues panel (**B**).

Supplementary Table 1: Top ranked hypomethylated or hypermethylated genes in HCC tumor compared with adjacent non-tumor tissues, reported in previous studies and in the present study. See Supplementary_Table_1
