# Supplementary material for: Epigenome-wide association study in hepatocellular carcinoma: Identification of stochastic epigenetic mutations through an innovative statistical approach
Source: Oncotarget. 2017 Apr 27;8(26):41890–902. doi: 10.18632/oncotarget.17462 (PMC5522036; doi:10.18632/oncotarget.17462)
Supplement: Supplementary file 2 [file oncotarget-08-41890-s002.docx]

| **Supplementary Table 1: Top ranked hypomethylated or hypermethylated genes in HCC tumor compared with adjacent non-tumor tissues, reported in previous studies and in the present study. Probes highlighted in blue have been detected in at least 2 studies.** | | | | | | | | | | | | | | | | | | | | | | | | | | | | | | | | | |
| --- | --- | --- | --- | --- | --- | --- | --- | --- | --- | --- | --- | --- | --- | --- | --- | --- | --- | --- | --- | --- | --- | --- | --- | --- | --- | --- | --- | --- | --- | --- | --- | --- | --- |
|  |  |  | |  | |  |  | |  |  | |  | |  |  |  |  |  |  | | |  | | | |  |  |  |  | |  | |  |
|  | **Hypermethylated genes** | | | | | | | | | | | | | | |  | **Hypomethylated genes** | | | | | | | | | | | | | | | | |
|  |  |  | |  | |  | |  |  | |  | |  |  |  |  |  |  |  | | |  | | | |  |  |  | |  | |  |  |
|  | **CpG-Site** | **Delta** | | **adj P Value** | | **Gene Symbol** | |  | **Mean Methylation Tumoral tissue** | | **Mean Methylation Peritumoral tissue** | | **Delta** | **adj P Value** |  |  |  | **CpG-Site** | **Delta** | | | **adj P Value** | | | | **Gene Symbol** |  | **Mean Methylation Tumoral tissue** | | **Mean Methylation Peritumoral tissue** | | **Delta** | **adj P Value** |
|  |  |  | |  | |  | |  |  | |  | |  |  |  |  |  |  |  | | |  | | | |  |  |  | |  | |  |  |
|  | **Jing Shen et al [6]; Top 20 significant CpG sites and genes within DMRs in HCC tumor tissues compared with adjacent non-tumor tissues Delta > 20%** | | | | | | |  | **Present Study** | | | | | |  |  |  | **Jing Shen et a [6]; Top 20 significant CpG sites and genes within DMRs in HCC tumor tissues compared with adjacent non-tumor tissues Delta > 20%** | | | | | | | | |  | **Present Study** | | | | | |
|  | cg04917181 | | 0.37 | | 4.89E-29 | TSPYL5 | |  | 0.55 | 0.21 | | 0.34 | | 1.09E-25 |  |  |  | cg11356247 | | | -0.3751 | | | 1.45E-26 | PTPRN2 | |  | 0.53 | 0.91 | | -0.38 | | 1.17E-31 |
|  | cg19429281 | | 0.51 | | 9.96E-29 | ZNF702P | |  | 0.64 | 0.16 | | 0.48 | | 8.74E-28 |  |  |  | cg23328109 | | | -0.2309 | | | 3.22E-26 | REXO1L2P | |  | 0.49 | 0.71 | | -0.22 | | 1.05E-32 |
|  | cg06445348 | | 0.46 | | 1.18E-28 | ILDR2 | |  | 0.65 | 0.17 | | 0.49 | | 4.74E-22 |  |  |  | cg22740796 | | | -0.5061 | | | 5.48E-26 |  | |  | 0.16 | 0.67 | | -0.51 | | 8.57E-28 |
|  | cg25622366 | | 0.49 | | 1.58E-28 | OTX1 | |  | 0.58 | 0.07 | | 0.52 | | 2.05E-29 |  |  |  | cg00084798 | | | -0.3316 | | | 7.41E-26 |  | |  | 0.42 | 0.74 | | -0.32 | | 1.94E-28 |
|  | cg03192598 | | 0.45 | | 2.97E-28 |  | |  | 0.67 | 0.23 | | 0.45 | | 3.25E-28 |  |  |  | cg06552160 | | | -0.3386 | | | 1.26E-25 | MYT1L | |  | 0.27 | 0.66 | | -0.39 | | 1.86E-27 |
|  | cg18267049 | | 0.42 | | 9.50E-28 |  | |  | 0.66 | 0.27 | | 0.40 | | 1.62E-25 |  |  |  | cg00217080 | | | -0.4936 | | | 2.06E-25 |  | |  | 0.30 | 0.82 | | -0.52 | | 1.42E-28 |
|  | cg16601494 | | 0.51 | | 1.23E-27 | C1orf70 | |  | 0.65 | 0.13 | | 0.52 | | 4.45E-28 |  |  |  | cg26032412 | | | -0.2495 | | | 4.01E-25 |  | |  | 0.26 | 0.53 | | -0.27 | | 6.99E-26 |
|  | cg13879483 | | 0.52 | | 1.26E-27 | USP44 | |  | 0.71 | 0.14 | | 0.57 | | 5.83E-28 |  |  |  | cg26986989 | | | -0.3231 | | | 4.26E-25 |  | |  | 0.26 | 0.53 | | -0.27 | | 6.99E-26 |
|  | cg22195627 | | 0.41 | | 1.98E-27 |  | |  | 0.68 | 0.30 | | 0.38 | | 1.83E-27 |  |  |  | cg07834841 | | | -0.4485 | | | 5.34E-25 |  | |  | 0.33 | 0.81 | | -0.47 | | 9.49E-28 |
|  | cg15487867 | | 0.52 | | 2.00E-27 | C1orf70 | |  | 0.56 | 0.04 | | 0.52 | | 7.68E-29 |  |  |  | cg08892613 | | | -0.4835 | | | 5.80E-25 |  | |  | 0.29 | 0.83 | | -0.54 | | 3.88E-28 |
|  | cg07835424 | | 0.43 | | 2.05E-27 |  | |  | 0.63 | 0.18 | | 0.44 | | 5.08E-27 |  |  |  | cg25225379 | | | -0.5112 | | | 6.11E-25 |  | |  | 0.23 | 0.76 | | -0.53 | | 5.59E-28 |
|  | cg13546935 | | 0.48 | | 4.10E-27 | SPDYA | |  | 0.64 | 0.14 | | 0.50 | | 5.67E-27 |  |  |  | cg09050058 | | | -0.3247 | | | 8.00E-25 |  | |  | 0.24 | 0.65 | | -0.42 | | 1.35E-25 |
|  | cg00346208 | | 0.35 | | 6.58E-27 | VWA5B1 | |  | 0.60 | 0.24 | | 0.35 | | 5.31E-30 |  |  |  | cg00887902 | | | -0.2687 | | | 8.32E-25 | XKR4 | |  | 0.25 | 0.56 | | -0.31 | | 2.96E-26 |
|  | cg15157455 | | 0.24 | | 1.73E-26 | HIST2H2BF | |  | 0.46 | 0.23 | | 0.23 | | 1.92E-26 |  |  |  | cg05767404 | | | -0.3033 | | | 2.83E-24 | C1orf150 | |  | 0.25 | 0.64 | | -0.39 | | 6.06E-26 |
|  | cg18081940 | | 0.39 | | 2.30E-26 | TDRD10 | |  | 0.63 | 0.26 | | 0.38 | | 7.14E-25 |  |  |  | cg13822911 | | | -0.3542 | | | 2.89E-24 |  | |  | 0.36 | 0.80 | | -0.44 | | 1.31E-27 |
|  | cg17694795 | | 0.35 | | 6.53E-26 |  | |  | 0.62 | 0.24 | | 0.38 | | 5.57E-28 |  |  |  | cg27104173 | | | -0.5479 | | | 3.28E-24 | PTPRN2 | |  | 0.34 | 0.93 | | -0.58 | | 2.69E-30 |
|  | cg06537894 | | 0.47 | | 7.40E-26 | MAST1 | |  | 0.60 | 0.12 | | 0.48 | | 2.70E-26 |  |  |  | cg20189761 | | | -0.2138 | | | 4.10E-24 | REXO1L2P | |  | 0.53 | 0.76 | | -0.23 | | 1.09E-33 |
|  | cg16657538 | | 0.49 | | 9.94E-26 | ZNF397OS | |  | 0.63 | 0.10 | | 0.53 | | 2.37E-29 |  |  |  | cg09120035 | | | -0.3666 | | | 4.12E-24 | CYP11B1 | |  | 0.37 | 0.82 | | -0.45 | | 2.55E-29 |
|  | cg00458878 | | 0.34 | | 1.23E-25 |  | |  | 0.54 | 0.16 | | 0.38 | | 1.72E-27 |  |  |  | cg00159780 | | | -0.2355 | | | 5.17E-24 | REXO1L2P | |  | 0.50 | 0.74 | | -0.24 | | 3.53E-32 |
|  | cg03679755 | | 0.4 | | 1.41E-25 |  | |  | 0.59 | 0.18 | | 0.41 | | 1.33E-26 |  |  |  | cg16158575 | | | -0.3199 | | | 1.11E-23 |  | |  | 0.38 | 0.78 | | -0.39 | | 4.76E-28 |
|  |  | |  | |  |  | |  |  |  | |  | |  |  |  |  |  | | |  | | |  |  | |  |  |  | |  | |  |
|  |  | |  | |  |  | |  |  | | | | | |  |  |  |  | | | | | | | | |  |  | | | | | |
|  |  | |  | |  |  | |  |  |  | |  | |  |  |  |  |  |  | | |  | | | |  |  |  |  | |  | |  |
|  | **Jing Shen et a [7]; Top 20 ranked hypermethylated genes** | | | | | | |  | **Present Study** | | | | | |  |  |  | **Jing Shen et a [7]; Top 20 ranked hypomethylated genes** | | | | | | | | |  | **Present Study** | | | | | |
|  | cg05684891 | | 0.4 | | 3.58E-17 | DAB2IP | |  | 0.57 | 0.20 | | 0.37 | | 3.40E-25 |  |  |  | cg21643045 | | | −0.32 | | | 1.7E-14 | | CCL20 |  | 0.50 | 0.84 | | -0.34 | | 2.43E-20 |
|  | cg14310034 | | 0.41 | | 5.36E-16 | BMP4 | |  | 0.51 | 0.11 | | 0.40 | | 3.58E-23 |  |  |  | cg11314684 | | | −0.15 | | | 9.34E-13 | | AKT3 |  | 0.15 | 0.48 | | -0.33 | | 6.32E-26 |
|  | cg12680609 | | 0.39 | | 3.25E-15 | ZFP41 | |  |  |  | |  | |  |  |  |  | cg01772980 | | | −0.27 | | | 2.28E-12 | | SCGB1D1 |  | 0.34 | 0.67 | | -0.33 | | 1.63E-24 |
|  | cg04786857 | | 0.36 | | 1.79E-14 | SPDY1 | |  | 0.50 | 0.12 | | 0.38 | | 1.53E-25 |  |  |  | cg24765446 | | | −0.28 | | | 3.42E-12 | | WFDC6 |  | 0.37 | 0.79 | | -0.42 | | 3.07E-25 |
|  | cg09099744 | | 0.42 | | 8.69E-14 | CDKN2A | |  |  |  | |  | |  |  |  |  | cg08886154 | | | −0.19 | | | 5.19E-12 | | PAX4 |  | 0.28 | 0.57 | | -0.30 | | 2.34E-23 |
|  | cg15747595 | | 0.25 | | 7.56E-13 | TSPYL5 | |  | 0.70 | 0.44 | | 0.26 | | 2.65E-21 |  |  |  | cg25462303 | | | −0.15 | | | 7.92E-12 | | GCET2 |  | 0.15 | 0.47 | | -0.33 | | 2.31E-28 |
|  | cg24432073 | | 0.35 | | 8.03E-13 | CDKL2 | |  | 0.58 | 0.07 | | 0.50 | | 3.25E-27 |  |  |  | cg04995095 | | | −0.22 | | | 8.87E-12 | | CD300E |  | 0.29 | 0.69 | | -0.40 | | 1.49E-24 |
|  | cg21790626 | | 0.43 | | 1.14E-12 | ZNF154 | |  | 0.49 | 0.04 | | 0.45 | | 2.54E-29 |  |  |  | cg04574507 | | | −0.25 | | | 9.89E-12 | | CD1B |  | 0.33 | 0.81 | | -0.48 | | 1.72E-27 |
|  | cg03975694 | | 0.3 | | 2.03E-12 | ZNF540 | |  | 0.59 | 0.25 | | 0.34 | | 1.41E-21 |  |  |  | cg03602500 | | | −0.26 | | | 1.37E-11 | | FLJ00060 |  | 0.42 | 0.73 | | -0.32 | | 4.90E-23 |
|  | cg00891278 | | 0.29 | | 5.07E-12 | CCDC37 | |  | 0.55 | 0.23 | | 0.32 | | 8.84E-22 |  |  |  | cg25119415 | | | −0.24 | | | 2.42E-11 | | MNDA |  | 0.25 | 0.65 | | -0.40 | | 5.51E-27 |
|  | cg11377136 | | 0.27 | | 5.18E-12 | PKDREJ | |  | 0.62 | 0.24 | | 0.38 | | 7.97E-29 |  |  |  | cg12200412 | | | −0.12 | | | 2.42E-11 | | CD1E |  | 0.23 | 0.44 | | -0.21 | | 2.86E-22 |
|  | cg08441806 | | 0.24 | | 2.77E-11 | NKX6-2 | |  | 0.52 | 0.25 | | 0.27 | | 1.03E-25 |  |  |  | cg09120035 | | | −0.31 | | | 2.59E-11 | | CYP11B1 |  | 0.37 | 0.82 | | -0.45 | | 2.55E-29 |
|  | cg15868302 | | 0.15 | | 2.88E-11 | FOXD2 | |  | - | - | | - | | - |  |  |  | cg02764897 | | | −0.20 | | | 2.84E-11 | | KRTAP13-1 |  | 0.33 | 0.69 | | -0.36 | | 3.04E-25 |
|  | cg06914598 | | 0.16 | | 4.22E-11 | RBAK | |  | - | - | | - | | - |  |  |  | cg01144251 | | | −0.25 | | | 2.95E-11 | | KLK9 |  | 0.40 | 0.68 | | -0.28 | | 2.71E-22 |
|  | cg25509184 | | 0.28 | | 4.23E-11 | CFTR | |  | 0.54 | 0.26 | | 0.28 | | 1.83E-14 |  |  |  | cg25564800 | | | −0.05 | | | 3.8E-11 | | KPNA1 |  | 0.06 | 0.17 | | -0.11 | | 2.47E-25 |
|  | cg07922606 | | 0.07 | | 2.38E-10 | HIST1H3E | |  | 0.85 | 0.77 | | 0.09 | | 1.17E-17 |  |  |  | cg18780284 | | | −0.26 | | | 4.44E-11 | | SPRR1B |  | 0.35 | 0.78 | | -0.43 | | 1.54E-26 |
|  | cg25340403 | | 0.14 | | 2.4E-10 | LYPD3 | |  | 0.71 | 0.58 | | 0.14 | | 1.51E-14 |  |  |  | cg04505023 | | | −0.30 | | | 4.82E-11 | | SPRR1A |  | 0.14 | 0.26 | | -0.12 | | 1.24E-13 |
|  | cg23391785 | | 0.36 | | 2.75E-10 | DNM3 | |  | 0.56 | 0.09 | | 0.47 | | 6.05E-27 |  |  |  | cg13615963 | | | −0.09 | | | 7.2E-11 | | CCR6 |  | 0.35 | 0.69 | | -0.34 | | 1.16E-26 |
|  | cg21554552 | | 0.21 | | 3.45E-10 | RASSF1 | |  | 0.66 | 0.31 | | 0.35 | | 4.52E-27 |  |  |  | cg13897627 | | | −0.22 | | | 1.05E-10 | | FLJ44674 |  | 0.38 | 0.79 | | -0.41 | | 1.19E-23 |
|  | cg27409364 | | 0.23 | | 3.7E-10 | KCNC1 | |  | - | - | | - | | - |  |  |  | cg06353345 | | | −0.23 | | | 1.06E-10 | | OR51B4 |  | - | - | | - | | - |
|  |  | |  | |  |  | |  |  |  | |  | |  |  |  |  |  | | |  | | |  | |  |  |  |  | |  | |  |
|  |  | |  | |  |  | |  |  | | | | | |  |  |  |  | | |  | | |  | |  |  |  | | | | | |
|  |  | |  | |  |  | |  |  |  | |  | |  |  |  |  |  | | |  | | |  | |  |  |  |  | |  | |  |
|  | **Song MA et a [8]; Top 20 ranked hypermethylated genes** | | | | | | |  | **Present Study** | | | | | |  |  |  | **Song MA et a [8]; Top 20 ranked hypomethylated genes** | | | | | | | | |  | **Present Study** | | | | | |
|  | cg01566592 | | 0.55 | | 3.60E-08 | RIMS2 | |  | 0.66 | 0.07 | | 0.59 | | 2.72E-27 |  |  |  | cg02714192 | | | -0.6 | | | 4.80E-06 | | NFATC1 |  | 0.41 | 0.92 | | -0.52 | | 4.53E-21 |
|  | cg08703872 | | 0.54 | | 2.50E-05 | C2CD4D | |  | 0.74 | 0.18 | | 0.56 | | 3.61E-23 |  |  |  | cg14715697 | | | -0.59 | | | 2.30E-12 | | HRNBP3 |  | 0.20 | 0.83 | | -0.63 | | 5.34E-30 |
|  | cg20749741 | | 0.53 | | 2.00E-05 | ACP1, | |  | 0.66 | 0.11 | | 0.54 | | 1.13E-21 |  |  |  | cg17366808 | | | -0.57 | | | 1.00E-11 | | C15orf60 |  | 0.43 | 0.93 | | -0.50 | | 1.52E-29 |
|  | cg19809499 | | 0.51 | | 9.00E-10 | FOXE3c | |  | 0.58 | 0.08 | | 0.50 | | 1.39E-25 |  |  |  | cg02310286 | | | -0.56 | | | 1.70E-10 | | DCAF4L2 |  | 0.39 | 0.90 | | -0.51 | | 3.57E-25 |
|  | cg23246885 | | 0.51 | | 3.40E-08 | FZD7 | |  | 0.67 | 0.12 | | 0.55 | | 1.30E-27 |  |  |  | cg11498870 | | | -0.54 | | | 4.20E-10 | | DCAF4L2 |  | 0.36 | 0.93 | | -0.56 | | 8.42E-31 |
|  | cg16657538 | | 0.51 | | 1.60E-08 | ZNF397OSb | |  | 0.63 | 0.10 | | 0.53 | | 2.37E-29 |  |  |  | cg21588562 | | | -0.53 | | | 6.90E-12 | | FTMT |  | 0.36 | 0.90 | | -0.54 | | 1.74E-28 |
|  | cg25945732 | | 0.5 | | 1.00E-05 | ACP1, | |  | 0.58 | 0.07 | | 0.51 | | 8.21E-23 |  |  |  | cg10480461 | | | -0.52 | | | 3.70E-08 | | C15orf60 |  | 0.48 | 0.95 | | -0.47 | | 4.30E-29 |
|  | cg13204512 | | 0.49 | | 2.20E-08 | RNF135 | |  | 0.53 | 0.04 | | 0.49 | | 1.77E-28 |  |  |  | cg13078134 | | | -0.51 | | | 1.90E-08 | | MARCH_1 |  | 0.44 | 0.95 | | -0.51 | | 2.11E-29 |
|  | cg24425838 | | 0.49 | | 3.20E-05 | C2CD4D | |  | 0.71 | 0.20 | | 0.51 | | 4.77E-23 |  |  |  | cg21181391 | | | -0.5 | | | 1.50E-14 | | DCAF4L2 |  | 0.36 | 0.90 | | -0.54 | | 3.98E-29 |
|  | cg05022673 | | 0.48 | | 1.70E-09 | BEND6 | |  | 0.64 | 0.11 | | 0.53 | | 5.42E-25 |  |  |  | cg26523649 | | | -0.47 | | | 5.30E-05 | | ARHGAP8 |  | 0.55 | 0.94 | | -0.38 | | 1.86E-19 |
|  | cg19306047 | | 0.47 | | 1.70E-03 | LPAR2c | |  | 0.48 | 0.03 | | 0.46 | | 8.86E-24 |  |  |  | cg19740859 | | | -0.46 | | | 6.90E-14 | | MYT1L |  | 0.40 | 0.88 | | -0.48 | | 7.12E-29 |
|  | cg12296772 | | 0.47 | | 1.90E-09 | MTMR7 | |  | 0.70 | 0.24 | | 0.46 | | 3.68E-29 |  |  |  | cg26598649 | | | -0.46 | | | 1.50E-14 | | DLGAP1 |  | 0.45 | 0.94 | | -0.49 | | 2.38E-27 |
|  | cg10523966 | | 0.47 | | 7.20E-04 | BMP8A | |  | 0.52 | 0.05 | | 0.46 | | 6.31E-23 |  |  |  | cg02432860 | | | -0.46 | | | 1.20E-05 | | GAB4 |  | 0.45 | 0.88 | | -0.44 | | 5.63E-23 |
|  | cg26440289 | | 0.46 | | 1.70E-05 | SNX31c | |  | 0.68 | 0.12 | | 0.56 | | 1.23E-26 |  |  |  | cg05945275 | | | -0.45 | | | 5.10E-06 | | GAB4 |  | 0.52 | 0.89 | | -0.37 | | 1.77E-20 |
|  | cg13096208 | | 0.46 | | 4.20E-05 | ST8SIA3 | |  | 0.56 | 0.08 | | 0.48 | | 1.17E-27 |  |  |  | cg05777962 | | | -0.45 | | | 3.70E-11 | | OR2B11 |  | 0.40 | 0.79 | | -0.39 | | 3.84E-28 |
|  | cg08162372 | | 0.46 | | 7.20E-04 | BMP4 | |  | 0.62 | 0.09 | | 0.53 | | 8.54E-26 |  |  |  | cg26681770 | | | -0.45 | | | 5.00E-03 | | PMEPA1 |  | 0.33 | 0.72 | | -0.39 | | 4.10E-17 |
|  | cg21790626 | | 0.45 | | 1.40E-04 | ZNF154 | |  | 0.49 | 0.04 | | 0.45 | | 2.54E-29 |  |  |  | cg01548777 | | | -0.44 | | | 3.40E-05 | | ZBTB46 |  | 0.47 | 0.84 | | -0.37 | | 3.99E-23 |
|  | cg03468349 | | 0.45 | | 5.60E-05 | ACP1, | |  | 0.53 | 0.05 | | 0.48 | | 8.65E-24 |  |  |  | cg14422922 | | | -0.44 | | | 3.10E-10 | | OR2B11 |  | 0.41 | 0.88 | | -0.47 | | 1.07E-26 |
|  | cg03585419 | | 0.45 | | 2.40E-05 | MYADM | |  | 0.50 | 0.07 | | 0.43 | | 2.06E-22 |  |  |  | cg01972576 | | | -0.44 | | | 4.00E-10 | | GML |  | 0.36 | 0.79 | | -0.44 | | 3.64E-26 |
|  | cg02339682 | | 0.45 | | 1.10E-08 | DST | |  | 0.64 | 0.11 | | 0.53 | | 1.98E-26 |  |  |  | cg06792448 | | | -0.44 | | | 3.70E-03 | | RTL1 |  | 0.47 | 0.92 | | -0.45 | | 1.69E-20 |
|  |  | |  | |  |  | |  |  |  | |  | |  |  |  |  |  | | |  | | |  | |  |  |  |  | |  | |  |
|  |  | |  | |  |  | |  |  | | | | | |  |  |  |  | | |  | | |  | |  |  |  | | | | | |
|  |  | |  | |  |  | |  |  |  | |  | |  |  |  |  |  | | |  | | |  | |  |  |  |  | |  | |  |
|  | **Neumann O et a [9]; list of the top hypermethylated genes with a median methylation difference > 0.45** | | | | | | |  | **Present Study** | | | | | |  |  |  | **Neumann O et a [9]; list of the top hypomethylated genes with a median methylation difference at least -0.40** | | | | | | | | |  | **Present Study** | | | | | |
|  | cg09053680 | | 0.62 | | <0.001 | UTF1 | |  | 0.55 | 0.13 | | 0.42 | | 3.01E-22 |  |  |  | cg06806711 | | -0.46 | | | <0.001 | | | MS4A1 |  | 0.32 | 0.64 | | -0.32 | | 5.28E-18 |
|  | cg25720804 | | 0.6 | | <0.001 | TLX3 | |  | 0.53 | 0.17 | | 0.36 | | 1.89E-19 |  |  |  | cg25856811 | | -0.46 | | | <0.001 | | | SPRR3 |  | 0.22 | 0.54 | | -0.31 | | 7.38E-26 |
|  | cg26521404 | | 0.6 | | <0.001 | HOXA9 | |  | 0.42 | 0.12 | | 0.30 | | 4.24E-12 |  |  |  | cg09120035 | | -0.46 | | | <0.001 | | | CYP11B1 |  | 0.37 | 0.82 | | -0.45 | | 2.55E-29 |
|  | cg04034767 | | 0.59 | | <0.001 | GRASP | |  | 0.56 | 0.04 | | 0.52 | | 5.39E-31 |  |  |  | cg06627364 | | -0.45 | | | <0.001 | | | MGC4677 |  | - | - | | - | | - |
|  | cg22881914 | | 0.57 | | <0.001 | NID2 | |  | 0.58 | 0.22 | | 0.36 | | 1.80E-13 |  |  |  | cg04505023 | | -0.44 | | | <0.001 | | | SPRR1A |  | 0.35 | 0.78 | | -0.43 | | 1.54E-26 |
|  | cg18815943 | | 0.54 | | <0.001 | FOXE3 | |  | 0.49 | 0.09 | | 0.40 | | 8.70E-29 |  |  |  | cg17725968 | | -0.43 | | | <0.001 | | | PDHA2 |  | 0.40 | 0.86 | | -0.46 | | 4.03E-25 |
|  | cg22375192 | | 0.54 | | <0.001 | IGF1R | |  | 0.54 | 0.07 | | 0.46 | | 1.11E-22 |  |  |  | cg11009736 | | -0.43 | | | <0.001 | | | MARCO |  | 0.20 | 0.61 | | -0.41 | | 3.03E-24 |
|  | cg08097882 | | 0.52 | | <0.001 | POU4F1 | |  | 0.53 | 0.16 | | 0.36 | | 9.06E-22 |  |  |  | cg15320474 | | -0.43 | | | <0.001 | | | UBD |  | 0.54 | 0.77 | | -0.23 | | 2.48E-10 |
|  | cg02440177 | | 0.52 | | <0.001 | ZNF702 | |  | 0.48 | 0.18 | | 0.30 | | 6.47E-18 |  |  |  | cg18780284 | | -0.42 | | | <0.001 | | | SPRR1B |  | - | - | | - | | - |
|  | cg18536148 | | 0.52 | | <0.001 | TBX4 | |  | 0.60 | 0.34 | | 0.26 | | 1.76E-13 |  |  |  | cg23595927 | | -0.41 | | | <0.001 | | | MYL5 |  | 0.45 | 0.69 | | -0.24 | | 2.02E-18 |
|  | cg04797323 | | 0.51 | | <0.001 | SOCS2 | |  | 0.52 | 0.21 | | 0.31 | | 3.46E-12 |  |  |  | cg18675600 | | -0.41 | | | <0.001 | | | PTP4A3 |  | 0.44 | 0.85 | | -0.40 | | 1.99E-20 |
|  | cg23391785 | | 0.51 | | <0.001 | DNM3 | |  | 0.56 | 0.09 | | 0.47 | | 6.05E-27 |  |  |  | cg08878744 | | -0.41 | | | <0.001 | | | LCE1B |  | 0.25 | 0.54 | | -0.28 | | 3.84E-24 |
|  | cg08668790 | | 0.5 | | <0.001 | ZNF154 | |  | 0.55 | 0.15 | | 0.39 | | 1.26E-19 |  |  |  | cg07592353 | | -0.4 | | | <0.001 | | | GABRA6 |  | 0.47 | 0.90 | | -0.43 | | 5.16E-25 |
|  | cg15191648 | | 0.5 | | <0.001 | SALL3 | |  | 0.39 | 0.19 | | 0.20 | | 1.73E-11 |  |  |  | cg08763351 | | -0.4 | | | <0.001 | | | SPRR4 |  | 0.53 | 0.92 | | -0.39 | | 8.73E-22 |
|  | cg13801416 | | 0.5 | | <0.001 | AKR1B1 | |  | 0.45 | 0.08 | | 0.37 | | 9.24E-25 |  |  |  | cg10501065 | | -0.4 | | | <0.001 | | | IGF2AS |  | 0.38 | 0.55 | | -0.17 | | 5.12E-07 |
|  | cg06377278 | | 0.5 | | <0.001 | RUNX3 | |  | 0.44 | 0.03 | | 0.41 | | 6.20E-22 |  |  |  |  | |  | | |  | | |  |  |  |  | |  | |  |
|  | cg09099744 | | 0.49 | | <0.001 | CDKN2A | |  | - | - | | - | | - |  |  |  |  | |  | | |  | | |  |  |  |  | |  | |  |
|  | cg00489401 | | 0.49 | | <0.001 | FLT4 | |  | 0.58 | 0.29 | | 0.29 | | 1.19E-13 |  |  |  |  | |  | | |  | | |  |  |  |  | |  | |  |
|  | cg06291867 | | 0.47 | | <0.001 | HTR7 | |  | 0.55 | 0.26 | | 0.30 | | 1.11E-23 |  |  |  |  | |  | | |  | | |  |  |  |  | |  | |  |
|  | cg02755525 | | 0.47 | | <0.001 | NETO2 | |  | 0.52 | 0.07 | | 0.45 | | 2.43E-25 |  |  |  |  | |  | | |  | | |  |  |  |  | |  | |  |
|  | cg07533148 | | 0.47 | | <0.001 | TRIM58 | |  | 0.35 | 0.05 | | 0.30 | | 1.68E-17 |  |  |  |  | |  | | |  | | |  |  |  |  | |  | |  |
|  | cg09260089 | | 0.47 | | <0.001 | NKX6-2 | |  | 0.62 | 0.17 | | 0.45 | | 2.46E-24 |  |  |  |  | |  | | |  | | |  |  |  |  | |  | |  |
|  | cg05684891 | | 0.47 | | <0.001 | DAB2IP | |  | 0.57 | 0.20 | | 0.37 | | 3.40E-25 |  |  |  |  | |  | | |  | | |  |  |  |  | |  | |  |
|  | cg21870884 | | 0.46 | | <0.001 | GPR25 | |  | 0.51 | 0.34 | | 0.18 | | 1.82E-14 |  |  |  |  | |  | | |  | | |  |  |  |  | |  | |  |
|  | cg15520279 | | 0.46 | | <0.001 | HOXD8 | |  | 0.49 | 0.20 | | 0.29 | | 1.26E-15 |  |  |  |  | |  | | |  | | |  |  |  |  | |  | |  |
|  | cg15433631 | | 0.45 | | <0.001 | IRX2 | |  | - | - | | - | | - |  |  |  |  | |  | | |  | | |  |  |  |  | |  | |  |
|  |  | |  | |  |  | |  |  |  | |  | |  |  |  |  |  | |  | | |  | | |  |  |  |  | |  | |  |
